# From Compromise to Burnout: Understanding Potential Pathways Linking Career Compromise and Burnout Among Novice Nurses via Latent Profile Analysis and Mediation Analysis

**DOI:** 10.1155/jonm/8245497

**Published:** 2026-10-28

**Authors:** Hongli Zhang, Xiaojuan Feng, Yacui Yuan, Zhentian Wu, Yuhan Wei, Qiaoying Yang, Yanting Han, Ka Li

**Affiliations:** ^1^ West China School of Nursing Sichuan University/West China Hospital Sichuan University Chengdu Sichuan China scu.edu.cn; ^2^ Medicine and Engineering Interdisciplinary Research Laboratory of Nursing & Materials West China Hospital Sichuan University/West China School of Nursing Sichuan University Chengdu Sichuan China scu.edu.cn; ^3^ Xi’an Weiyang Baoshihua Xinglongyuan Hospital Xi’an Shaanxi China; ^4^ Department of General Surgery Breast Center West China Hospital Sichuan University/West China School of Nursing Sichuan University Chengdu Sichuan China scu.edu.cn; ^5^ The Second Affiliated Hospital of Air Force Medical University Xi’an Shaanxi China fmmu.edu.cn; ^6^ The Affiliated Hospital of Qingdao University Qingdao Shandong China qdu.edu.cn

**Keywords:** burnout, career compromise, latent profile analysis, mediation analysis, novice nurses, occupational identity

## Abstract

**Background:**

Retention of novice nurses is a key strategy for alleviating nursing shortages and ensuring the sustainability of the nursing profession. During the transition to clinical practice, novice nurses face unique challenges and may experience career compromise when attempting to reconcile their career expectations with workplace realities. Career compromise has been associated with burnout and even turnover. However, career compromise among novice nurses has received insufficient research attention. This study aimed to identify profiles of career compromise among novice nurses and examine the role of occupational identity in the relationship between different career compromise profiles and burnout, thereby providing insights for improving novice nurse retention.

**Methods:**

A cross‐sectional survey was conducted from August to December 2025. A total of 570 novice nurses completed an online questionnaire that collected demographic information and included the Career Compromise Scale, Occupational Identity Scale, and Burnout Scale. Latent profile analysis was used to identify career compromise profiles. Univariate and logistic regression analyses were conducted to examine factors statistically associated with profile membership. Finally, the potential statistical indirect association involving occupational identity between career compromise profiles and burnout was examined.

**Results:**

Three career compromise profiles were identified: a low career compromise profile (*n* = 211, 37.0%), a moderate career compromise profile (*n* = 169, 29.6%), and a high career compromise profile (*n* = 190, 33.3%). Gender, marital status, educational level, monthly income, employment form, working time every day (hours), interested in nursing, and having the thought to resign were statistically associated with profile membership. Analysis of statistical indirect associations showed that, compared with the low career compromise profile, both the moderate and high career compromise profiles were associated with lower occupational identity and higher burnout.

**Conclusions:**

Novice nurses exhibited career compromise profiles reflecting different levels of career compromise, with approximately two‐thirds experiencing moderate or high levels of career compromise. Occupational identity partially accounted for the observed association between career compromise profiles and burnout. Occupational identity may partially account for the observed association between career compromise profiles and burnout, with a statistically significant indirect association. Identifying sociodemographic and work‐related factors associated with career compromise and providing tailored support for nurses with different career compromise profiles may help inform interventions addressing career compromise and burnout among novice nurses. However, the effectiveness of such interventions requires evaluation in longitudinal and intervention studies.

**Implications for Nursing Management:**

Assessing subgroups of novice nurses according to their career compromise profiles can inform hypothesis‐generating and potentially targeted support strategies and promote continuous improvement. Supporting early career adaptation and considering occupational identity in support strategies may be relevant to burnout and novice nurse retention. The potential value of such approaches should be evaluated in prospective and intervention studies before implementation.

## 1. Introduction

The nursing shortage remains a major challenge for healthcare systems worldwide. It is estimated that there will be a global shortfall of 4.5 million nurses by 2030. More importantly, nurse turnover rates remain persistently high, ranging from 8.0% to 36.6% globally [1, 2]. However, turnover among novice nurses is even more concerning. Research has shown that 60% of nurses leave their first nursing position within their first year of employment [3]. The turnover of novice nurses requires organizations to invest additional resources and incur additional costs in staff recruitment and training. High turnover can also undermine the morale of remaining nurses, who must cope with heavier workloads and an unstable work environment, thereby potentially compromising patient safety [4]. Therefore, retaining novice nurses is an important strategy for maintaining healthcare system stability and safeguarding public safety.

Novice nurses are generally defined as registered nurses with no more than 3 years of clinical work experience [5]. In China, novice nurses account for approximately 47% of frontline clinical nurses, and this proportion is increasing [6]. The transition from nursing student to practicing nurse is a critical period for novice nurses, during which they undergo role transition, develop early professional competencies, and establish career aspirations [7]. They are required to rapidly acquire knowledge and skills, integrate into clinical teams, assume responsibilities in highly demanding environments, and confront human suffering. Empirical studies have indicated that most novice nurses experience substantial work‐related stress, intense work demands, limited remuneration and career development opportunities, and public questioning of the value of the nursing profession; these challenges constitute major reasons for their turnover [8]. In a qualitative study of 16 Chinese novice nurses conducted by Waner Wu, some participants reported that work was not merely a means of financial compensation but also a process of self‐realization [9]. However, during the transition from nursing intern to clinical nurse, they perceived substantial differences between internship and actual clinical work in terms of workload, responsibility, and interpersonal relationships, resulting in a strong sense of discrepancy and discomfort. These experiences may widen the gap between their ideal career aspirations and actual work environments, diminish their career motivation, and weaken their positive expectations regarding future career development [10]. Such circumstances may be associated with career compromise.

Career compromise is an important concept in career development [11]. According to career development theories, career compromise refers to the process by which individuals adjust, lower, or relinquish their original career goals and expectations in response to perceived discrepancies between occupational realities and their career expectations or goals, in order to adapt to real‐world conditions [12]. Thus, the gap between an ideal career and a realistically attainable occupation represents an important context for the emergence of career compromise, whereas career compromise itself reflects the adjustments and concessions individuals make in response to this gap. Theoretically, career compromise emphasizes the cognitive and psychological process through which individuals adjust their original career goals and expectations when faced with practical constraints during career adaptation and development [13]. In other words, career compromise concerns how individuals manage the discrepancy between their “ideal career” and “actual career,” rather than their evaluation of their current job, subjective experiences of job stress, occupational identity, or behavioral intentions to leave the profession. For nurses, career compromise refers to the adjustments and concessions made when there is a substantial discrepancy between actual nursing work and their own expectations [14]. Career compromise is closely related to the career development of novice nurses. It is also an important occupational issue, as research suggests that career compromise may be associated with perceived self‐efficacy, occupational identity, and mental health [15]. Studies have found that higher levels of career compromise are associated with lower job satisfaction, weaker innovative behavior, and greater turnover intention and burnout [16, 17]. As the future nursing workforce, novice nurses may be particularly affected by career compromise in terms of their career adaptation and career outcomes. Nevertheless, little is known about career compromise among novice nurses, and research on its characteristics and associated factors and relationships in this population remains limited.

Persistent emotional and psychological stress in the workplace, manifested as depletion of emotional and physical resources, may develop into an occupational state known as burnout [18]. Burnout has been recognized by the World Health Organization (WHO) as an occupational phenomenon [19]. According to Maslach’s multidimensional model, burnout is defined as an occupational syndrome characterized by emotional exhaustion, depersonalization, and reduced personal accomplishment [20]. Nurses are at high risk of burnout, which has attracted widespread attention. A nationwide survey in China found that 33% of nurses reported high levels of fatigue and low efficiency, while the prevalence of high depersonalization among nurses ranged from 11% to 56% [21, 22]. Burnout adversely affects nurses’ health, work performance, and turnover and may even impair their ability to provide high‐quality care. Studies have shown that newly graduated nurses transitioning from school to clinical practice are more vulnerable to burnout and demonstrate stronger turnover intentions than their experienced colleagues [23]. In a study by Huan Ma, 52.46% of novice nurses reported high levels of burnout [24]. Research has also found that individuals with higher levels of career compromise report more burnout‐related manifestations, including reduced career expectations, work alienation, decreased occupational efficacy, and inadequate work productivity [25]. Collectively, these findings indicate an observed association between career compromise and burnout among novice nurses.

Occupational identity may partially account for the observed association between career compromise and burnout. Occupational identity refers to nurses’ recognition and acceptance of the goals, responsibilities, values, and social significance of the nursing profession. It reflects individuals’ positive evaluation of their occupational role and their sense of belonging to the profession [26]. A higher level of occupational identity has been associated with greater understanding of the meaning of nursing work, recognition of the value of nursing to patients and society, and greater occupational engagement and persistence, as well as lower levels of burnout [27]. Conversely, when actual work conditions fail to meet individuals’ career expectations, they may adapt to reality by lowering their career goals or adjusting their career ideals. Career compromise, less positive appraisals of the value and future prospects of nursing, and lower occupational identity may therefore be statistically interrelated [28]. In addition, a systematic review of 15 studies reported that professional identity development programs may have potential relevance to burnout prevention and therefore warrant further evaluation [29]. Multiple studies have reported that stronger occupational identity is associated with lower levels of burnout among nurses [30, 31].

Conservation of Resources (COR) theory provides a useful perspective for understanding the observed relationships among career compromise, occupational identity, and burnout [32]. This theory proposes that individuals are intrinsically motivated to acquire, maintain, and protect valued resources. Psychological stress and negative outcomes are likely to arise when individuals experience resource loss, perceive their resources to be threatened, or continuously invest resources without obtaining the expected returns. Resources include not only material conditions and social support but also psychological resources, such as a sense of control, perceived competence, a sense of occupational meaning, and positive self‐evaluation [33]. For novice nurses, the transition from nursing student to clinical nurse is accompanied by increased job demands, greater professional responsibilities, and adjustments in career expectations [34]. When there is a substantial discrepancy between actual nursing work and their original career expectations, nurses may adapt to reality by lowering or adjusting their career goals; this process is manifested as career compromise [35]. Although career compromise may reflect a reduced perceived discrepancy between career ideals and current circumstances, a higher level of career compromise may also be associated with perceptions that resources such as career development opportunities, occupational control, perceived competence, and a sense of occupational value are threatened or depleted.

Occupational identity refers to nurses’ recognition and acceptance of the value of nursing, their professional roles, and their sense of belonging to the profession. It is associated with novice nurses’ sense of occupational meaning, value, and belonging and may therefore represent an important psychological resource [36]. A higher degree of career compromise may be associated with less positive appraisals of the value and future prospects of the nursing profession and with lower occupational identity. Lower occupational identity may co‐occur with fewer perceived psychological resources for understanding the meaning of their work, sustaining occupational engagement, and coping with clinical work demands. Lower occupational identity may also be associated with burnout manifestations, such as emotional exhaustion, work alienation, and reduced personal accomplishment. Therefore, career compromise may be associated with burnout among novice nurses, with occupational identity potentially accounting for part of this statistical association. Different overall levels of career compromise may be associated with differences in perceived psychological resources and may therefore be differentially associated with occupational identity and burnout. Overall, novice nurses with different levels of career compromise may differ in occupational identity and burnout, and occupational identity may partially account for the observed statistical association between career compromise and burnout.

As an essential reserve force within the nursing profession, novice nurses are critical to the sustainable development and stability of the nursing workforce [37]. Career compromise may reflect tensions between career ideals and workplace realities during novice nurses’ career adaptation and may be associated with perceived depletion of occupational resources and diminished expectations for career development. These associations may have implications for workforce stability and patient safety. Therefore, examining the characteristics of career compromise among novice nurses and its potential relationships with occupational identity and burnout is of considerable importance. Previous studies have indicated that some nurses experience high levels of career compromise, whereas others report moderate overall levels. However, existing studies have predominantly used variable‐centered analytical approaches, treating nurses as a homogeneous population and paying limited attention to individual differences in career compromise [38]. Such approaches may fail to identify potential subgroups of novice nurses with different overall levels of career compromise. Latent profile analysis (LPA) is a person‐centered analytical approach that classifies individuals with similar characteristics into latent profiles reflecting different overall levels based on their response patterns across observed indicators of career compromise [39]. LPA provides a more nuanced understanding of heterogeneity in career compromise among novice nurses and enables the identification of groups with different overall levels of career compromise. These profiles may provide a basis for generating hypotheses about differentiated support needs, but should not be interpreted as validated categories.

In summary, a person‐centered approach may help identify subgroups of novice nurses with distinct career compromise profiles and examine their relationships with occupational identity and burnout. The findings of this study may contribute to hypothesis generation regarding potential support needs among novice nurses and may inform future research on career compromise, retention, and nursing workforce sustainability. Accordingly, this study proposes the following hypotheses:

 Hypothesis 1. Career compromise among novice nurses comprises several meaningful latent profiles.
 Hypothesis 2. Novice nurses with different career compromise profiles will differ in occupational identity and burnout, and a statistically significant indirect association involving occupational identity may be observed between career compromise profiles and burnout. The strength of this statistical indirect association may vary across profiles.

The specific aims of this study were to

1. explore whether latent profiles reflecting different overall levels of career compromise exist among novice nurses,
2. compare demographic characteristics and occupational factors across the identified subgroups,
3. identify factors statistically associated with membership in subgroups with different overall levels of career compromise, thereby helping to characterize novice nurses who may have different support needs, and
4. examine the statistical indirect association involving occupational identity in the relationship between latent career compromise profiles and burnout.

The findings of this study may generate hypotheses for future research on addressing career compromise during the early stages of novice nurses’ careers and supporting the long‐term sustainability of the nursing profession.

## 2. Materials and Methods

### 2.1. Study Design

This study used a cross‐sectional design. The reporting and presentation of the study findings followed the Strengthening the Reporting of Observational Studies in Epidemiology (STROBE) statement [40].

### 2.2. Participants

From August to December 2025, a convenience sample of 630 novice nurses was recruited from seven tertiary hospitals across four provinces in China: Shaanxi, Shandong, Sichuan, and Hebei. The inclusion criteria were (1) having graduated within the past 3 years and (2) registered nurses employed in clinical settings. Nurses in internship or standardized residency training programs, those unwilling to participate, and those who were not on duty during the survey period were excluded.

Before the survey was conducted, permission was obtained from the participating hospitals. With the assistance of nursing managers, electronic questionnaires were distributed to eligible nurses. The first page of the questionnaire provided detailed information about the study purpose, significance, and completion requirements. Participants could access the questionnaire only after selecting the “informed and agreed” option. They were informed that they could withdraw from the survey at any stage without any consequences.

### 2.3. Sample Size

LPA was the primary analytical method used in this study. Sample size requirements for LPA should consider the identification of latent classes, the stability of model estimation, and the number of participants in each latent class [41]. According to recommendations from relevant methodological studies, a sample size of more than 500 is generally required for LPA to avoid model nonconvergence or failure to identify small profiles. In addition, each profile should include at least 50 participants to ensure statistical reliability [42]. Therefore, 630 questionnaires were distributed, and 570 valid questionnaires were ultimately obtained.

### 2.4. Measures

#### 2.4.1. Sociodemographic Characteristics

Sociodemographic and occupational characteristics were collected using a self‐designed questionnaire. These variables included gender, age, marital status, educational level, income (CNY/month), interested in nursing, working time every day (hours), having the thought to resign, and employment form.

#### 2.4.2. Career Compromise Scale

Career compromise was measured using the Career Compromise Scale developed by Weng Qingxiong [43]. The scale comprises three dimensions: Development Opportunity Compromise (four items; e.g., “When considering whether to enter your current position/occupation, please carefully recall your satisfaction with future promotion opportunities”); Career Matching Compromise (four items; e.g., “When considering whether to enter your current position/occupation, please carefully recall the extent to which it was consistent with your career ideals and career goals”), and Social Expectations Compromise (four items; e.g., “When considering whether to enter your current job/occupation, please carefully recall your satisfaction with the social status associated with your employer and position”). Each item is rated on a 5‐point Likert scale ranging from 1 (“Strongly Disagree”) to 5 (“Strongly Agree”). In the present study, the Cronbach’s *α* coefficient for the scale was 0.974.

#### 2.4.3. Occupational Identity Scale

Occupational identity was assessed using the Occupational Identity Scale developed by Tyler and McCallum (1998) [44]. The scale consists of 10 items, including “An important part of my self‐image is based on the work that I currently do” and “My career trajectory plays an important role in understanding who I am.” Items are rated on a 5‐point Likert scale from 1 (“Strongly Disagree”) to 5 (“Strongly Agree”), with higher total scores indicating a higher level of occupational identity. The scale has been widely validated and has demonstrated satisfactory reliability and validity [45]. In the present study, Cronbach’s *α* was 0.986.

#### 2.4.4. Burnout Scale

Burnout was assessed using the Chinese version of the Maslach Burnout Inventory–General Survey (MBI–GS), originally developed by Maslach et al. [46], and revised in Chinese by Li Chaoping [47]. The scale comprises three dimensions: Emotional Exhaustion (five items; e.g., “I feel tired when I get up in the morning and have to face another day on the job”); Cynicism (four items; e.g., “Since I started this job, I have become less interested in my work”); and Professional Efficacy (six items; e.g., “I can effectively solve the problems that arise in my work”). Items are rated on a 7‐point Likert scale ranging from “never” to “very frequently.” The professional efficacy dimension is reverse‐scored, and higher total scores indicate more severe burnout. In the present study, Cronbach’s *α* was 0.856.

### 2.5. Data Collection

Data were collected using an online questionnaire platform. Before distributing the questionnaire, the researchers contacted nursing managers at each participating hospital. With the assistance of these managers, electronic questionnaires were distributed through WeChat, a social media platform, to nurses who met the eligibility criteria. The principles of informed consent and privacy protection were strictly followed. Participants could access the survey only after indicating that they had read and agreed to the informed consent statement. All responses were collected anonymously. Participants were informed of their right to withdraw from the study at any time without any adverse consequences. To ensure data validity and reliability, each participant received appropriate instructions throughout the survey process. Only one questionnaire submission was permitted from each IP address, and questionnaires in which the same response option was selected for all items were excluded. A total of 630 questionnaires were distributed. Of these, 40 participants withdrew during the survey, and 20 questionnaires were excluded because identical responses were provided for all items. Ultimately, 570 valid questionnaires were retained, yielding an effective response rate of 90.48%.

### 2.6. Data Analysis

All statistical analyses were conducted using IBM SPSS 26.0 and Mplus 8.3. First, descriptive statistics were performed using IBM SPSS 26.0. Continuous variables were presented as means and standard deviations, whereas categorical variables were described using frequencies and percentages. All tests were two‐tailed, and *p* < 0.05 was considered statistically significant.

Second, LPA was conducted using Mplus 8.3 to identify latent profiles reflecting different response patterns and overall levels of career compromise based on the 12 items of the Career Compromise Scale among novice nurses. The 12 items were used as observed indicators, and participants were classified according to their response patterns across these items. Models with one to five latent profiles were fitted sequentially. The optimal model was determined based on model fit indices, class sizes, model parsimony, and substantive interpretability. The model fit indices included the Akaike information criterion (AIC), Bayesian information criterion (BIC), and sample‐size‐adjusted Bayesian information criterion (aBIC), with lower values indicating a better model fit. The Lo–Mendell–Rubin adjusted likelihood ratio test (LMRT) and bootstrap likelihood ratio test (BLRT) were used to further evaluate and compare model fit. A *p* value of < 0.05 indicated that the k‐class model fit the data significantly better than the (*k* − 1) class model. Entropy values range from 0 to 1 and were used to assess the accuracy of profile classification; an entropy value of ≥ 0.80 indicates high classification accuracy [48, 49]. Compared with simple grouping based on total career compromise scores, LPA simultaneously uses information from all 12 items to identify latent populations in a data‐driven manner and estimate individuals’ probabilities of membership in different profiles.

Third, factors associated with membership in different latent career compromise profiles among novice nurses were explored. The latent classes generated in the second step were used as the grouping variable. Chi‐square tests were used for categorical variables, and analysis of variance was used for continuous variables to identify relevant sociodemographic and occupational characteristics. Variables that were statistically significant in the univariate analyses (*p* < 0.05) were examined for multicollinearity and subsequently entered into a multinomial logistic regression model, with Profile 1 as the reference category. This analysis was used to estimate factors statistically associated with membership in the different career compromise profiles. A significance level of *p* < 0.05 was used for both univariate and multinomial logistic regression analyses.

Fourth, PROCESS Version 3.5, Model 4, was used to examine the statistical indirect association involving occupational identity (M, mediator) in the observed association between career compromise profiles (X, independent variable) and burnout (Y, dependent variable). Because career compromise profiles constituted a categorical variable, the profiles were represented using appropriate dummy variables, with Profile 1 as the reference category. The bootstrap procedure was performed with 5000 resamples, and 95% bias‐corrected confidence intervals (CIs) were calculated. A statistical indirect association was considered significant if the 95% bias‐corrected CI for the indirect effect did not include zero [50].

### 2.7. Ethical Considerations

This study was conducted in accordance with the ethical principles of the Declaration of Helsinki [51]. All participants were informed that they could withdraw from the study at any time. All survey data were kept strictly confidential and were used solely for the purposes of this study. The research protocol was approved by the Clinical Research Ethics Committee of Xi’an Weiyang Baoshihua Xinglongyuan Hospital (Approval No. XACHQ‐YJ‐25‐068).

## 3. Results

### 3.1. Descriptive Statistics

A total of 570 participants were included in this study. Of the participants, 93.5% were female, 66.9% were aged ≤ 26 years, 62.6% were married, 74.2% had a bachelor’s degree, 72.1% had a monthly income of CNY 3001–6,000, 57.5% reported moderate interest in nursing, 54.2% worked 8–10 h per day, 77.0% had no thoughts of resigning, and 74.9% were employed under the contract system. Other sociodemographic and occupational characteristics are presented in Table 1.

**TABLE 1:** Participant characteristics.

Variable	Overall, *n* = 570	Low career compromise, *n* = 211 (37.0%)	Moderate career compromise, *n* = 169 (29.6%)	High career compromise, *n* = 190 (33.3%)	*p* value
Gender					0.001^a^
Male	37 (6.5%)	24 (11.4%)	8 (4.7%)	5 (2.6%)	
Female	533 (93.5%)	187 (88.6%)	161 (95.3%)	185 (97.4%)	
Age					0.884^a^
≤ 23	171 (30.0%)	64 (30.3%)	53 (31.4%)	54 (28.4%)	
24–26	193 (33.9%)	77 (36.5%)	56 (33.1%)	73 (38.4%)	
≥ 27	206 (36.1%)	70 (33.2%)	60 (35.5%)	63 (33.2%)	
Marital status					< 0.001^a^
Unmarried	213 (37.4%)	79 (37.4%)	81 (47.9%)	53 (27.9%)	
Married	357 (62.6%)	132 (62.6%)	88 (52.1%)	137 (72.1%)	
Educational level					< 0.001^a^
Junior college	117 (20.5%)	36 (17.1%)	24 (14.2%)	57 (30.0%)	
Bachelor	423 (74.2%)	158 (74.9%)	135 (79.9%)	130 (68.4%)	
Master or above	30 (5.3%)	17 (8.1%)	10 (5.9%)	3 (1.6%)	
Income (CNY/month)					< 0.001^a^
≤ 3000	54 (9.5%)	28 (13.3%)	19 (11.2%)	7 (3.7%)	
3001–6000	411 (72.1%)	148 (70.1%)	135 (79.9%)	128 (67.4%)	
≥ 6001	105 (18.4%)	35 (16.6%)	15 (8.9%)	55 (28.9%)	
Interested in nursing					< 0.001^a^
No	140 (24.6%)	63 (29.9%)	46 (27.2%)	31 (16.3%)	
Moderate	328 (57.5%)	96 (45.5%)	100 (59.2%)	132 (69.5%)	
Yes	102 (17.9%)	52 (24.6%)	23 (13.6%)	27 (14.2%)	
Working time every day (hours)					< 0.001^a^
≤ 7	146 (25.6%)	106 (50.2%)	30 (17.8%)	10 (5.3%)	
8–10	309 (54.2%)	87 (41.2%)	119 (70.4%)	103 (54.2%)	
≥ 11	115 (20.2%)	18 (8.5%)	20 (11.8%)	77 (40.5%)	
Having the thought to resign					< 0.001^a^
Yes	131 (23.0%)	23 (10.9%)	20 (11.8%)	88 (46.3%)	
No	439 (77.0%)	188 (89.1%)	149 (88.2%)	102 (53.7%)	
Employment form					< 0.001^a^
Contact system	427 (74.9%)	137 (64.9%)	118 (69.8%)	172 (90.5%)	
Permanent	83 (14.6%)	39 (18.5%)	30 (17.8%)	14 (7.4%)	
Other	60 (10.5%)	35 (16.6%)	21 (12.4%)	4 (2.1%)	
Occupational identity	39.12 ± 9.23	44.14 ± 8.17	37.37 ± 7.89	35.11 ± 8.94	< 0.001^b^
Burnout	42.61 ± 18.10	30.46 ± 13.76	48.43 ± 16.22	50.91 ± 16.70	< 0.001^b^
Emotional exhaustion	15.8 ± 8.40	10.91 ± 5.89	17.82 ± 7.04	19.42 ± 9.30	< 0.001^b^
Cynicism	10.88 ± 6.83	6.58 ± 3.93	12.04 ± 5.82	14.61 ± 7.59	< 0.001^b^
Reduced personal accomplishment	16.26 ± 10.11	12.63 ± 9.35	18.8 ± 9.32	18.03 ± 10.48	< 0.001^b^

^a^Pearson’s chi‐squared test.

^b^Analysis of variance (ANOVA).

### 3.2. Latent Profile Analysis

As shown in Table 2, the AIC, BIC, and aBIC generally decreased as the number of latent profiles increased, indicating that adding profiles could improve model fit to some extent. Although the four‐ and five‐profile models had lower AIC, BIC, and aBIC values, the BLRT results for these models were not significant (*p* = 0.109 and *p* = 0.112, respectively), suggesting that adding a fourth or fifth profile did not significantly improve model fit. In addition, both the four‐ and five‐profile models contained small classes, accounting for 5.4% and 5.1% of the sample, respectively. Such small subgroups may result in unstable parameter estimates and reduce the reproducibility of the model in other samples, indicating limited classification validity and practical applicability [52]. In contrast, the three‐profile model was identified as the optimal model, with satisfactory fit indices: AIC = 20177.258, BIC = 20394.540, and aBIC = 20235.812. Furthermore, the LMRT (*p* < 0.001) and BLRT (*p* = 0.008) values were significant, indicating that the three‐profile solution was statistically supported relative to the two‐profile solution. The entropy value was 0.969, reflecting high classification accuracy. The smallest profile included 169 participants, accounting for 29.6% of the total sample, supporting the stability and interpretability of the three‐profile solution. Taking into account the model fit indices, LMRT and BLRT results, classification accuracy, class sample sizes, model parsimony, and substantive interpretability, the three‐profile model was selected for describing heterogeneity in overall levels of career compromise in this study (Figure 1).

**TABLE 2:** Fit indices and group sizes of the latent profile models.

Indices	Model				
	1‐profile	2‐profile	3‐profile	4‐profile	5‐profile
Fit statistics					
AIC	27290.885	21883.104	20177.258	19444.057	18859.214
BIC	27395.151	22043.893	20394.540	19717.832	19189.483
aBIC	27318.916	21926.435	20235.812	19517.835	18948.217
Entropy	—	0.967	0.969	0.984	0.981
BLRT	—	0.020	0.008	0.109	0.112
LMR	—	< 0.001	< 0.001	< 0.001	< 0.001
Group sizes (%)					
C1	570 (100.0%)	313 (54.9%)	211 (37.0%)	207 (36.2%)	203 (35.6%)
C2	—	257 (45.1%)	169 (29.6%)	155 (27.3%)	29 (5.1%)
C3	—	—	190 (33.3%)	31 (5.4%)	128 (22.4%)
C4	—	—	—	177 (31.1%)	141 (24.8%)
C5	—	—	—	—	69 (12.1%)

*Note:* LMR, Lo–Mendell–Rubin likelihood ratio test.

Abbreviations: AIC, Akaike information criterion; aBIC, adjusted Bayesian information criterion; BIC, Bayesian information criterion; BLRT, bootstrap likelihood ratio test.

**FIGURE 1:**
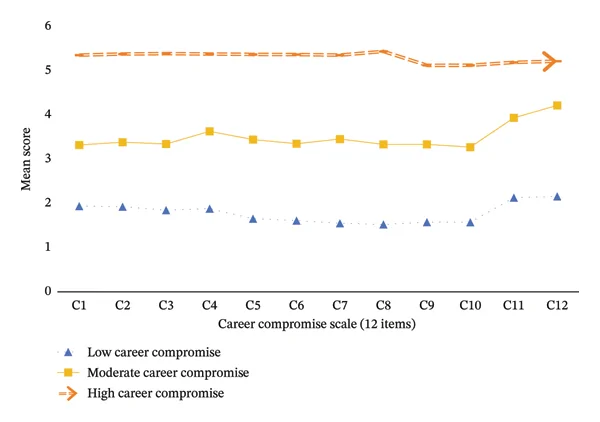
Profile plot of the three career compromise profiles.

Table 3 presents the mean scores for the three dimensions of career compromise among novice nurses. In the three‐profile solution, novice nurses were classified into three latent profiles that differed primarily in their overall levels of career compromise. Analysis of variance showed statistically significant differences in total career compromise scores and scores for all three dimensions across the profiles (*p* < 0.001). Further least significant difference (LSD) post hoc analyses indicated that all pairwise differences between the groups were statistically significant (*p* < 0.001). Across each dimension of the Career Compromise Scale, the mean scores followed the same pattern: Class 3 > Class 2 > Class 1. Accordingly, and to reflect this predominantly quantitative gradient, Class 3, Class 2, and Class 1 were labeled the high, moderate, and low career compromise profiles, respectively (Class 3, *n* = 190, 33.3%; Class 2, *n* = 169, 29.6%; and Class 1, *n* = 211, 37.0%). The average posterior probabilities calculated according to the most‐likely class membership approach were 99.2%, 99.2%, and 97.2% for Classes 1, 2, and 3, respectively, indicating a high degree of certainty in participants’ profile membership.

**TABLE 3:** Career compromise of new nurses in different profiles.

	*N*	Career compromise	Development opportunity compromise	Occupational fit compromise	Social expectation compromise
Low career compromise	211	21.18 ± 6.43	7.54 ± 3.66	6.28 ± 2.21	7.36 ± 3.05
Moderate career compromise	169	41.72 ± 5.67	13.6 ± 3.88	13.51 ± 3.48	14.61 ± 3.74
High career compromise	190	63.39 ± 6.69	21.38 ± 2.49	21.41 ± 2.3	20.61 ± 3.84
*F*		2241.036	836.27	1596.991	705.986
*p*		< 0.001	< 0.001	< 0.001	< 0.001
LSD		C1 < C2 < C3	C1 < C2 < C3	C1 < C2 < C3	C1 < C2 < C3

### 3.3. Univariate Analysis and Ordinal Multinomial Regression

The univariate analysis showed significant differences among the three career compromise profiles in gender, marital status, educational level, income (CNY/month), interested in nursing, working time every day (hours), having the thought to resign, and employment form. Significant differences were also observed in occupational identity and burnout across the three profiles (*p* < 0.05). Specifically, participants in the high career compromise profile reported the lowest occupational identity and the highest burnout. Detailed results are presented in Table 1.

Multicollinearity diagnostics showed that the variance inflation factors (VIFs) for the independent variables ranged from 1.024 to 1.133, while tolerance values ranged from 0.882 to 0.977. All values were within acceptable ranges (VIF < 10 and tolerance > 0.10) [53]. Variables that were statistically significant in the univariate analyses were entered into the multinomial logistic regression model, with the low career compromise profile as the reference group (Table 4). Compared with the low career compromise profile, being unmarried, having a monthly income of CNY 3001–6,000, and reporting moderate interest in nursing were statistically associated with membership in the moderate career compromise profile. Compared with the low career compromise profile, having a junior college education and being employed under the contract system were statistically associated with membership in the high career compromise profile.

**TABLE 4:** Multivariate logistic regression results of three profiles of career compromise.

Variable	Moderate career compromise		High career compromise	
	OR (95% CI)	*p* value	OR (95% CI)	*p* value
Gender (ref.: female)				
Male	0.31 (0.13, 0.78)	**0.012**	0.27 (0.08, 0.87)	**0.029**
Age (ref.: ≥ 27)				
≤ 23	1.02 (0.58, 1.81)	0.934	0.69 (0.35, 1.37)	0.291
24–26	0.85 (0.49, 1.45)	0.542	1.15 (0.61, 2.14)	0.668
Marital status (ref.: married)				
Unmarried	1.82 (1.11, 2.97)	**0.018**	0.53 (0.30, 0.96)	**0.035**
Educational level (ref.: master or above)				
Junior college	0.80 (0.28, 2.32)	0.677	9.76 (1.77, 53.96)	**0.009**
Bachelor	1.07 (0.42, 2.73)	0.886	4.67 (0.91, 23.91)	0.064
Income (CNY/month) (ref.: ≥ 6001)				
≤ 3000	2.23 (0.88, 5.66)	0.093	0.29 (0.09, 0.93)	**0.038**
3001–6000	2.22 (1.08, 4.57)	**0.030**	0.48 (0.24, 0.94)	**0.032**
Interested in Nursing (ref.: yes)				
No	1.11 (0.55, 2.23)	0.779	0.69 (0.29, 1.65)	0.402
Moderate	1.92 (1.04, 3.57)	**0.039**	2.35 (1.13, 4.90)	**0.023**
Working time every day (hours) (ref.: ≥ 11)				
≤ 7	0.24 (0.11, 0.55)	**0.001**	0.03 (0.01, 0.08)	**< 0.001**
8–10	1.39 (0.67, 2.89)	0.382	0.37 (0.18, 0.73)	**0.005**
Having the thought to resign (ref.: yes)				
No	1.06 (0.53, 2.12)	0.868	0.19 (0.10, 0.37)	**< 0.001**
Employment form (ref.: other)				
Contact system	1.52 (0.78, 3.02)	0.230	30.65 (9.44, 99.49)	**< 0.001**
Permanent	1.87 (0.82, 4.23)	0.136	9.70 (2.50, 37.60)	**0.001**

*Note:* The low career compromise profile served as the reference group. Bold: *p* < 0.05. ref.: reference.

Abbreviations: CI, confidence interval; OR, odds ratio.

### 3.4. Statistical Indirect Associations Involving Occupational Identity in the Relationship Between Career Compromise Profiles and Burnout

All demographic and occupational confounding variables were included in the model for adjustment. To examine the statistical indirect associations involving occupational identity, the LPA‐derived career compromise profiles were dummy‐coded, with the low career compromise profile serving as the reference group. An indirect association model was specified with career compromise profile membership as the independent variable, occupational identity as the mediator, and burnout as the dependent variable. Compared with the low career compromise reference group, both the moderate and high career compromise profiles were significantly and negatively associated with occupational identity (*β* = −6.91, *p* < 0.001, *β* = −9.63, *p* < 0.001, respectively). After occupational identity was included in the model, the associations of the moderate and high career compromise profiles with burnout remained significant (*β* = 13.89, *p* < 0.001, *β* = 14.32, *p* < 0.001, respectively). In addition, occupational identity was significantly and negatively associated with burnout (*β* = −0.44, *p* < 0.001).

The bootstrap results showed that the 95% confidence intervals for both statistical indirect associations did not include zero, indicating that the indirect associations involving occupational identity were statistically significant. Specifically, the statistical indirect association involving occupational identity between the moderate career compromise profile and burnout was 3.01, with 95% CI (1.69, 4.61). The statistical indirect association involving occupational identity between the high career compromise profile and burnout was 4.19, with a 95% CI of 2.46 to 6.18. The proportions of the observed associations statistically accounted for by occupational identity were 17.82% and 22.64%, respectively. The overall model explained 31.80% of the variance in burnout (*R* ^2^ = 0.318). Detailed results are shown in Figure 2 and Tables 5 and 6.

**FIGURE 2:**
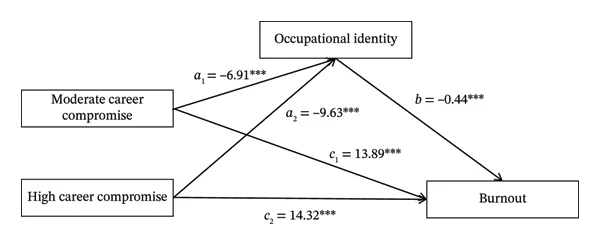
Mediation model of the associations between career compromise profiles, occupational identity, and burnout among novice nurses. Note: The moderate and high career compromise profiles were compared with the low career compromise profile, which served as the reference group. Occupational identity was examined as the mediator, and burnout was the outcome variable. The coefficients shown are unstandardized coefficients from the covariate‐adjusted mediation model. ^∗∗∗^ *p* < 0.001. The low career compromise profile was used as the reference group. The indirect effects were 3.01 (95% bootstrap CI [1.69, 4.61]) for the moderate profile and 4.19 (95% bootstrap CI [2.46, 6.18]) for the high profile, based on 5000 bootstrap samples.

**TABLE 5:** Regression paths of career compromise profiles, occupational identity, and burnout.

Outcome variables	Independent variables	Unadjusted analysis					Adjusted analysis					*R* ^2^
		*β*	SE	*t*	*p*	(LLCI, ULCI)	*β*	SE	*t*	*p*	(LLCI, ULCI)	
OI	Moderate career compromise	−6.78	0.86	−7.86	< 0.001	(−8.47, −5.08)	−6.91	0.89	−7.80	< 0.001	(−8.65, −5.17)	0.224
	High career compromise	−9.04	0.84	−10.81	< 0.001	(−10.68, −7.39)	−9.63	1.03	−9.33	< 0.001	(−11.66, −7.61)	

Burnout	Moderate career compromise	15.06	1.64	9.16	< 0.001	(11.83, 18.28)	13.89	1.72	8.08	< 0.001	(10.51, 17.26)	0.318
	High career compromise	16.56	1.66	9.97	< 0.001	(13.30, 19.82)	14.32	2.04	7.00	< 0.001	(10.30, 18.33)	
	OI	−0.43	0.08	−5.65	< 0.001	(−0.58, −0.28)	−0.44	0.08	−5.58	< 0.001	(−0.59, −0.28)	

*Note:* The low career compromise profile was set as the reference group. Unadjusted analysis did not control for demographic covariates, whereas adjusted analysis controlled for demographic covariates. Values in parentheses represent 95% confidence intervals. The *R* ^2^ values in the table correspond to the adjusted analysis.

Abbreviation: OI, occupational identity.

**TABLE 6:** Total, direct, and indirect effects of career compromise profiles on burnout.

Efects	Variables	Unadjusted analysis			Adjusted analysis		
		Estimate	SE	(LLCI, ULCI)	Estimate	SE	(LLCI, ULCI)
Total effect	Moderate career compromise	17.97	1.60	(14.82, 21.12)	16.89	1.68	(13.60, 20.18)
	High career compromise	20.45	1.55	(17.40, 23.50)	18.51	1.95	(14.67, 22.34)

Direct effect	Moderate career compromise	15.06	1.64	(11.83, 18.28)	13.89	1.72	(10.51, 17.26)
	High career compromise	16.56	1.66	(13.30, 19.82)	14.32	2.04	(10.30, 18.33)

Indirect effect	Moderate career compromise	2.91	0.71	(1.62, 4.40)	3.01	0.74	(1.69, 4.61)
	High career compromise	3.88	0.85	(2.30, 5.62)	4.19	0.93	(2.46, 6.18)

*Note:* Unadjusted analysis: covariates were not controlled; adjusted analysis: demographic variables were controlled. The low career compromise profile served as the reference group. The confidence intervals for the indirect effects in both profiles did not contain zero, indicating that the mediating effect of occupational identity was significant.

## 4. Discussion

This study used LPA to identify latent profiles of career compromise among novice nurses. The findings indicated that career compromise among novice nurses was not simply characterized by uniform differences across the same dimensions; rather, it comprised distinct patterns with varying levels of severity. The three profiles primarily reflected a gradient of overall career compromise, with differences in the magnitude rather than completely separate qualitative types of compromise. Our findings were consistent with the proposed hypotheses regarding profile differences and statistical associations. Previous studies have described nurses’ career compromise at the group level. Although these studies have provided valuable evidence, they may overlook individual differences in the overall levels and response patterns of career compromise. This limitation may reduce their practical relevance for improving career compromise among novice nurses. In contrast, LPA can address the question of which novice nurses demonstrate similar patterns of career compromise. Research on burnout among nurses has also gradually shifted from examining overall levels to identifying distinct patterns and combinations of individual characteristics [54, 55]. For example, Cao et al. used a person‐centered approach to examine different burnout profiles among Chinese nurses and further analyzed the roles of the nursing work environment and perceived quality of nursing care [56]. These findings suggest that nurses’ occupational psychological states exhibit substantial individual heterogeneity and that profile analysis has theoretical and practical value for identifying characteristics of different subgroups. This study identified three career compromise profiles: low, moderate, and high career compromise. The trends across the items were generally parallel among the three profiles, with between‐group differences primarily reflecting the overall degree of career compromise. Therefore, the heterogeneity identified in this study indicates that different groups of novice nurses may differ in their overall levels of career compromise, rather than representing three completely distinct qualitative types of career compromise. Compared with categorizing nurses according to arbitrarily defined cut‐off values based on total career compromise scores, LPA provides additional insights into response patterns across individual items and facilitates the identification of subgroups with different degrees of career compromise.

Based on LPA, we identified subgroups of career compromise among novice nurses, which represents an important finding of this study. The three profiles were labeled low career compromise (Class 1), moderate career compromise (Class 2), and high career compromise (Class 3), accounting for 37.0%, 29.6%, and 33.3% of the sample, respectively. From the perspective of COR theory, career development opportunities and organizational support may constitute important work‐related resources, whereas professional competence and occupational identity may serve as important personal resources for novice nurses [57]. When actual nursing work differs substantially from original career expectations, novice nurses may adapt to reality by adjusting their career goals or lowering their occupational expectations. This adaptation process may manifest as different overall levels of career compromise [58]. In the present study, the high career compromise profile accounted for approximately one‐third of the sample. This proportion describes the distribution within the present sample and should not be extrapolated directly to the broader population of novice nurses. Nevertheless, the finding suggests that career compromise may warrant attention during the early stages of nurses’ careers. Previous research has shown that novice nurses experience considerable stress while adapting to their professional roles and workplace culture. They may feel distrusted or undervalued; face continual criticism, evaluation, comparison, and blame; and experience negative emotions such as anxiety, fear, feelings of failure, and insecurity when their actual work does not align with their expectations. During this adaptation period, they may adjust or even abandon their original career goals [59, 60]. These experiences may be associated with different levels of career compromise among novice nurses.

This study further compared differences in general sociodemographic characteristics and occupational factors across the latent career compromise profiles. The findings showed that unmarried novice nurses, those with a lower monthly income, and those reporting moderate interest in nursing were more likely to be classified into the moderate career compromise profile. Ong et al. investigated nurses in different countries and found that, when managing dual roles, nurses often experience work–family conflict and compromise their career aspirations to prioritize family responsibilities, while neglecting their own self‐worth [61]. In addition, previous studies have indicated that income is associated with nurses’ job satisfaction, occupational identity, and intention to remain in the profession [62]. Novice nurses are at an early stage of their careers, during which their clinical experience and career development goals may not yet be fully established. When actual work content, workload, and occupational rewards differ from their original expectations, they may adapt to nursing work by lowering career expectations and adjusting career goals [63]. In particular, novice nurses with only moderate interest in nursing may have relatively lower occupational engagement and intrinsic work motivation. When confronted with substantial clinical pressures, they may be more likely to perceive inconsistencies between occupational realities and their original expectations, which may be associated with moderate levels of career compromise.

Having a junior college education and being employed under the contract system were statistically associated with membership in the high career compromise profile. Studies have shown that nurses with lower educational qualifications and those employed under contract arrangements tend to report higher levels of career compromise, which is consistent with the present findings [38]. Duygu Akbaş Uysal et al. found that higher educational attainment may be associated with professional knowledge, clinical decision‐making, and the ability to manage complex problems, thereby strengthening nurses’ sense of control at work and reducing uncertainty during career development [64]. In contrast, novice nurses with junior college education may face more constraints regarding continuing education, promotion opportunities, and professional development. When personal career expectations do not match actual development opportunities, they may be more likely to experience career‐related disappointment [65]. Furthermore, employment type may be associated with nurses’ occupational attitudes through differences in perceived job security and organizational support [66]. Novice nurses employed under the contract system may be more concerned about job stability, benefits, and promotion opportunities. When work demands do not correspond to occupational security and developmental returns, they may experience greater career uncertainty and, consequently, report higher levels of career compromise [67]. Overall, examining factors associated with different latent career compromise profiles may help characterize the circumstances associated with profile membership and generate hypotheses for future research on differentiated support needs. These findings should not be interpreted as providing a validated basis for identifying individual nurses as being at high risk of career compromise.

This study found differences in burnout levels among novice nurses across the latent career compromise profiles, with nurses experiencing higher levels of career compromise reporting relatively higher levels of burnout. This finding is consistent with the recent trend in nursing research of examining burnout from a person‐centered perspective [68]. Nurse burnout demonstrates substantial individual heterogeneity, and the nursing work environment and perceived quality of nursing care may be associated with different burnout patterns. Although this study did not further identify latent burnout profiles, the observed differences in burnout across career compromise profiles suggest that the association between career compromise and burnout may vary across groups of novice nurses. Nurse burnout is generally not caused by a single event but is the result of the long‐term accumulation of persistent and systemic work‐related stressors. Evidence suggests that nurse burnout is more closely related to chronic work stress and should not be attributed solely to a particular public health emergency [69]. For novice nurses, role transition, workload, night shift schedules, insufficient clinical competence, and uncertainty about career development may exacerbate the depletion of occupational resources [70, 71]. When career compromise occurs in work environments characterized by high workloads, limited support, or insufficient occupational rewards, individuals may be more susceptible to burnout.

Notably, the association between career compromise and burnout may also be related to novice nurses’ occupational identity. Higher levels of career compromise may be accompanied by a reduced sense of occupational value and professional belonging, and lower occupational identity may be associated with higher burnout. Occupational identity may therefore partially account for the observed association between career compromise and burnout. This study further examined the statistical indirect association involving occupational identity between latent career compromise profiles and burnout and found that this association was statistically significant. This finding suggests that differences in burnout among novice nurses with different career compromise profiles may be partially accounted for by occupational identity. Individuals with higher career compromise may hold less favorable evaluations of the value of nursing, their professional role, or their career prospects, thereby reporting lower occupational identity [72]. Lower occupational identity may be associated with a reduced ability to derive meaning, belonging, and a sense of value from nursing work, as well as fewer psychological resources when facing clinical work‐related stress, which may be related to greater vulnerability to burnout [73].

Occupational identity among novice nurses is gradually developed through professional socialization and clinical practice. During the initial transition into clinical practice, novice nurses need to redefine the social value, professional responsibilities, and role boundaries of nursing, while developing confidence in their own professional competence and career development through practical work experience [74]. If a substantial discrepancy exists between work realities and original career expectations, and nurses lack adequate organizational support and positive occupational experiences, their identification with the nursing profession may be negatively affected [75]. A high level of career compromise may reflect substantial adjustments to individuals’ original career goals. When accompanied by a diminished sense of occupational value or limited confidence in career development, career compromise may be associated with lower occupational identity and higher levels of burnout.

From the perspective of COR theory, occupational identity can be regarded as an important psychological resource. A strong occupational identity can help novice nurses affirm the value of nursing and their professional roles, thereby enhancing their sense of occupational meaning, belonging, and willingness to remain engaged in their work. When individuals gain a sense of value, professional growth, and social contribution from nursing, occupational identity may help preserve their psychological resources and enhance their ability to cope with clinical stress. Conversely, career compromise may be associated with greater uncertainty regarding the value and future prospects of nursing. This uncertainty may co‐occur with a weaker sense of occupational identity, which may in turn be associated with higher levels of burnout. However, the cross‐sectional design of this study does not permit conclusions regarding the temporal ordering or causality of these associations.

### 4.1. Limitations

This study has several limitations. First, the cross‐sectional design captured career compromise at only one point in time and precluded conclusions regarding temporal relationships or causality among career compromise, occupational identity, and burnout. Longitudinal studies are needed to examine changes in career compromise over time and to clarify the temporal relationships among these variables. Future studies could also use repeated measurements to examine whether occupational identity and burnout change alongside career compromise during the transition into clinical practice. Second, the sample was recruited from healthcare institutions in selected regions of China, which may limit the representativeness and generalizability of the findings. Future multicenter studies involving hospitals at different levels and from diverse regions are warranted. In addition, the use of convenience sampling may have introduced selection bias; therefore, the identified profile distribution should not be assumed to represent the distribution among novice nurses nationally. Third, this study examined occupational identity as the only hypothesized mediator and did not include other potentially relevant variables involved in indirect associations or moderating variables. Future research should incorporate additional individual, organizational, and work‐related factors to further clarify the temporal and potentially reciprocal relationships among career compromise, occupational identity, and burnout among novice nurses. Potentially relevant factors may include perceived organizational support, workload, career adaptability, professional self‐efficacy, social support, and work–family conflict. Fourth, the latent profiles were derived from the observed response patterns in this sample, and their reproducibility in other samples remains to be established. Future research should examine whether similar profiles can be replicated in independent samples and whether profile membership changes over time.

## 5. Conclusion

Using LPA, this study identified three career compromise profiles among novice nurses: low, moderate, and high career compromise. Approximately 62.9% of participants were classified into the moderate or high career compromise profiles. Several sociodemographic and work‐related characteristics were associated with profile membership, including gender, marital status, educational level, monthly income, interest in nursing, daily working hours, having the thought to resign, and employment form. Occupational identity and burnout also differed across the profiles, with the high career compromise profile showing lower occupational identity and higher burnout. Occupational identity may partially account for the observed association between career compromise profiles and burnout. These findings provide empirical evidence for stratified interventions by clinical nursing managers. Individualized support strategies should be developed for novice nurses with different levels of career compromise, with particular emphasis on addressing occupational identity and career compromise. Such strategies may help reduce burnout risk, stabilize the nursing workforce, and support the quality of clinical nursing services.

## 6. Implications for Nursing Management

As an emerging force within the nursing workforce, novice nurses are undergoing a critical transition into independent clinical practice. Career compromise is associated not only with novice nurses’ physical and psychological well‐being and intention to remain in nursing but also may be relevant to the quality of clinical nursing services and workforce stability. It has therefore become an important management concern. This study identified three latent profiles of career compromise among novice nurses—low, moderate, and high career compromise—and further examined factors associated with membership in these profiles. The findings may provide a basis for nursing managers to consider differentiated occupational support, subject to confirmation in longitudinal and intervention research.

For novice nurses in the high career compromise profile, managers should consider paying particular attention to nurses who report heavier workloads, thoughts of resigning, low occupational identity, or high burnout. Because these characteristics were identified as statistically associated factors or profile differences rather than causal risk factors, they should be used to guide supportive assessment rather than definitive risk classification. Nursing managers could use induction interviews, career adaptation assessments, and regular individual communication to understand specific difficulties related to workload, job adaptation, career development, organizational support, and intentions to resign. Case discussions and scenario simulations may help novice nurses identify high‐pressure work situations that are likely to trigger negative occupational experiences, such as sudden deterioration in patients’ conditions, unsuccessful resuscitation attempts, conflicts with patients or family members, ineffective teamwork, and continuous high‐intensity work. In addition, training in occupational value clarification, career planning, emotion recognition, cognitive reappraisal, and problem‐focused coping could be considered as components of a broader support program. Such training may help novice nurses better understand the pressures and challenges of clinical work and may support their responses to negative experiences associated with discrepancies between occupational expectations and actual work conditions. The effectiveness of these approaches should be evaluated in future intervention studies.

For nurses with low occupational identity, managers may strengthen their recognition of the value of nursing through sharing exemplary nursing practices, discussing patient recovery cases, providing feedback on nursing achievements, and offering career development guidance. For nurses who have developed thoughts of resigning, targeted retention interviews could be conducted to identify the specific sources of such thoughts. Depending on their circumstances, schedule adjustments, job adaptation support, career counseling, or psychological support could be provided.

For novice nurses classified into the moderate career compromise profile, management support could focus on occupational adaptation and on factors that may be associated with higher levels of career compromise. Managers should pay particular attention to nurses with low occupational identity and high levels of emotional exhaustion and cynicism. Through regular career adaptation assessments and group feedback, managers can identify difficulties related to clinical work, interpersonal communication, workload, and career development. Clinical scenario simulations, professional competency training, and task management training may help support novice nurses’ ability to organize work, manage time, and solve problems in high‐pressure clinical environments. At the same time, a peer‐support system involving experienced nurses, specialist nurses, and clinical preceptors could be established to provide clinical decision‐making guidance, emotional support, and practical assistance to novice nurses in the moderate career compromise profile. The feasibility and effectiveness of such profile‐sensitive support should be examined empirically.

For nurses in the low career compromise profile, managers may focus on maintaining positive occupational experiences and supporting continued professional development. Opportunities for mentoring, professional growth, recognition, and participation in clinical learning activities may help sustain engagement with the nursing profession.

Overall, nursing managers may consider developing multilevel support strategies informed by different career compromise profiles and their associated characteristics. The implementation of differentiated and adaptive nursing management measures may help address career compromise among novice nurses and support their occupational adaptation. Such measures may also contribute to nursing workforce stability. However, these implications should be regarded as practice‐oriented suggestions rather than evidence of proven intervention effects.

## Author Contributions

Hongli Zhang: conceptualization, methodology, validation, formal analysis, data curation, writing–original draft, and writing–review and editing. Xiaojuan Feng: methodology and formal analysis. Yacui Yuan: validation and data curation. Zhentian Wu: methodology and investigation. Qiaoying Yang: resources and investigation. Yanting Han: project administration, writing–review and editing, and funding acquisition. Ka Li: supervision, project administration, methodology, and conceptualization.

## Funding

The authors received no specific funding for this study.

## Disclosure

All authors have read and approved the final submitted version.

## Ethics Statement

This study was conducted in accordance with the ethical principles of the Declaration of Helsinki. All participants were informed that they could withdraw from the study at any time. All survey data were kept strictly confidential and were used solely for the purposes of this study. The research protocol was approved by the Clinical Research Ethics Committee of Xi’an Weiyang Baoshihua Xinglongyuan Hospital (Approval No. XACHQ‐YJ‐25‐068).

## Conflicts of Interest

The authors declare no conflicts of interest.

## Data Availability

The data that support the findings of this study are available upon request from the corresponding author. The data are not publicly available due to privacy or ethical restrictions.
